# Draft Genome Sequence of the Multidrug-Resistant Strain Pseudomonas aeruginosa PA291, Isolated from Cystic Fibrosis Sputum

**DOI:** 10.1128/MRA.00572-21

**Published:** 2021-09-09

**Authors:** Tiffany Luong, Ashley Schumann, Douglas Conrad, Dwayne Roach

**Affiliations:** a Department of Biology, San Diego State University, San Diego, California, USA; b Division of Pulmonary, Critical Care, and Sleep Medicine, Department of Medicine, University of California San Diego, La Jolla, California, USA; University of Arizona

## Abstract

Here, we report the genome sequence of PA291, a nonmucoid, multidrug-resistant strain of Pseudomonas aeruginosa isolated from cystic fibrosis sputum. Short reads were *de novo* assembled into 190 contigs and scaffold assembled to a length of 6.26 Mbp. PhiSpy predicts that PA291 is free of prophages.

## ANNOUNCEMENT

Pseudomonas aeruginosa causes difficult-to-treat and often multidrug-resistant (MDR) infections, which severely impacts individuals with cystic fibrosis (CF) (1, 2). CF patients are often required to take daily antibiotics, which promotes the development of chronic MDR infections (3). Phage therapy to combat MDR infections is under investigation (4, 5); in part, it requires both phenotypic and genomic characterization of the patient’s infectious isolate(s).

In this study, a bacterial colony from CF sputum (under UC San Diego Institutional Review Board-approved protocol number 160078) was cultured overnight on blood agar at 37°C, and matrix-assisted laser desorption ionization–time of flight (MALDI-TOF) mass spectrometry identified it as belonging to the species P. aeruginosa. The strain was named PA291 and found to have a nonmucoid phenotype, and a broth microdilution assay (performed by the UCSD Clinical Molecular Microbiology Laboratory following FDA/CLSI guidelines) revealed strong antibiotic resistance to ceftazidime (MIC > 16 μg/ml) and intermediate resistance to aztreonam (MIC, 4 μg/ml), gentamicin (MIC, 8 μg/ml), and meropenem (MIC, 4 μg/ml). Moreover, PA291 was lysed by phages PAK_P1, E215, and E217, which are under investigation to treat P. aeruginosa infections (5–7).

PA291 DNA extraction and purification was performed after overnight growth in Lennox broth (LB) at 37°C using the phenol-chloroform method (8). The DNA was sent to the Microbial Genome Sequencing Center (Pittsburgh, PA), which prepared an indexed paired-end library using the Illumina Nextera DNA Flex library prep kit and sequenced using the NextSeq 550 sequencer. A total of 4,939,438 reads were obtained, with a mean length of 134 bases. Quality control (QC) of FASTQ files was conducted using fastp v0.21.0 (9), and the short reads were *de novo* assembled using SPAdes v3.14.1 (10) into 190 contigs. The contigs were assembled using the scaffolder MeDuSa v1.6 (11) with the following reference genomes: PAO1 (GenBank accession number AE004091.2), W16407 (CP008869.2), VA-134 (CP013245.1), DVT779 (CP050330.1), SE5357 (CP054844.1), H47921 (CP008861.1), SE5443 (CP046405.1), PAO1161 (CP032126.1), PABL048 (CP039293.1), and PAC6 (CP053705.1). The quality and metrics were analyzed using QUAST v5.02 (12). All software was used with default settings. PA291 was found to have a length of 6.26 Mbp over 3 contigs and an *N*_50_ value of 6.16 Mbp. Its GC content is 66.55%. RAST (Rapid Annotation using Subsystems Technology) (13) predicted that PA291 carries 6,114 genes, including motility genes (*pulE/tfp*, *pilB*) and alginate biosynthesis genes (*alg*, *kinB*) (14). In addition, RAST, RGI v5.1.1, and CARD v3.1.1 (15) were used to identify 47 antibiotic resistance genes, including efflux pumps (*MATE* family, *opr*), a polymyxin resistance gene (*arnC*), and a fosfomycin resistance gene (*fosA*). PhiSpy v4.2.6 (16) did not predict that PA291 carried complete viral genomes (prophages).

In summary, we report the nearly complete genome sequence of a nonmucoid MDR P. aeruginosa strain isolated from CF sputum. The size of PA291 is consistent with that of other P. aeruginosa genomes, between 5.5 and 7 Mbp (17). This strain carries several antibiotic resistance, motility, and alginate biosynthesis genes commonly associated with strains isolated from chronic CF infections (18). Extensive antibiotic usage has negative health impacts and further promotes drug resistance. Given the PA291 MDR and lack of prophages, phage therapy may be a suitable alternative treatment.

### Data availability.

Data for P. aeruginosa PA291 can be found in the NCBI database under BioProject accession number PRJNA727612 and BioSample accession number SAMN19026471. The raw reads can be found in the Sequence Read Archive (SRA) under accession number SRR14436913. The RAST annotation of the genome is available on Zenodo (https://zenodo.org/record/5114517#.YPXHVehKguV). The genome sequence of P. aeruginosa PA291 was deposited in GenBank under accession number JAGYWJ000000000, where the Prokaryotic Genome Annotation Pipeline (PGAP) annotation is also available.
